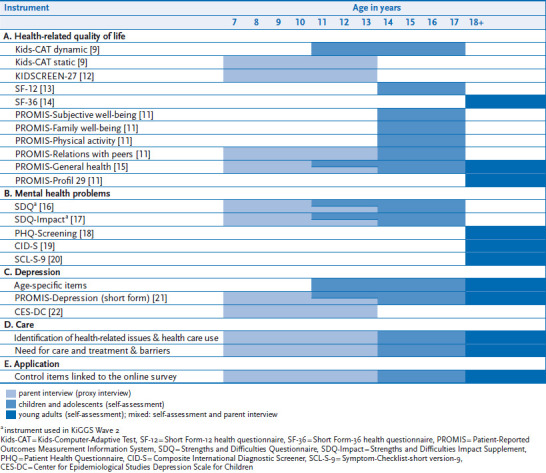# The BELLA study – the mental health module of KIGGS Wave 2

**DOI:** 10.17886/RKI-GBE-2017-109

**Published:** 2017-09-27

**Authors:** Fionna Klasen, Franziska Reiß, Christiane Otto, Anne-Catherine Haller, Ann-Katrin Meyrose, Dana Barthel, Ulrike Ravens-Sieberer

**Affiliations:** University Medical Center Hamburg-Eppendorf, Department of Child and Adolescent Psychiatry and Psychotherapy, Research Division Child Public Health, Hamburg

**Keywords:** BELLA STUDY, MENTAL HEALTH, QUALITY OF LIFE, HEALTH MONITORING, KIGGS

## Abstract

The BELLA study is the module on mental health and health-related quality of life within the German Health Interview and Examination Survey for Children and Adolescents (KiGGS). Baseline data collection took place together with KiGGS baseline data collection between 2003 and 2006. This article discusses the fourth follow-up of the BELLA study (BELLA Wave 4), which was surveyed between 2014 and 2017. The aims of the BELLA Wave 4 are to enable longitudinal analyses of health-related quality of life and mental health problems. Dynamic measurement instruments were used to enable a user-friendly and precise assessment of mental health among children, adolescents and young adults. The study’s participants were a sub-sample of around 3,500 KiGGS respondents aged 7 to 29 years. For the first time, in BELLA Wave 4 data were collected exclusively online. The BELLA study targeted both the parents of younger children (aged 7 to 13 years) and adolescents and young adults themselves (aged 11 years and above). Study instruments surveying mental health problems and the use of mental health care services were supplemented by a dynamic measurement tool in the form of a computer adaptive test (CAT) to record data on health-related quality of life.

## 1. Background and objective

The past century has seen significant changes to the challenges facing child health. While medical progress has greatly curbed the threat of infectious diseases, mental disorders such as depression and anxiety disorders are today among the most frequent illnesses affecting children and adolescents [1, 2]. Mental health disorders affect an estimated 20% of children and adolescents in Germany [1-3], and can lead to significant limitations for families, at school and impact a person’s wider social environment [4-6]. Moreover, mental health disorders in children and adolescents have a high risk of becoming chronic, and the development of comorbidities (accompanying diseases) is frequent in this group, which means that further mental disorders might develop [7]. This underlines the high public health relevance of mental health as an important factor in strengthening healthy childhood development and ensuring social participation. Furthermore, subjective well-being and quality of life are considered important aspects for modern concepts of health, especially in terms of prevention and intervention [8].

The BELLA study on mental health is conducted by professors Ulrike Ravens-Sieberer and Fionna Klasen at the University Medical Center Hamburg-Eppendorf’s Child Public Health department and has been from its start one of the supplementary modules of the German National Health Interview and Examination Survey among Children and Adolescents (KiGGS). The module strives for a more in-depth study of mental health and health-related quality of life among children and adolescents in Germany. BELLA Wave 4 has been designed as both a cohort and cross-sectional study, which means that respondents from previous waves (BELLA cohort) as well as a sample of new participants (cross-sectional) is surveyed. This approach provides not only a representative assessment of mental health in German-speaking children and adolescents, but also affords insights into the development of mental health over time, as eleven years have now passed since the baseline study.

**BELLA study Wave 4**

Fourth wave of the survey on the mental health and health-related quality of life of children, adolescents and young adults in Germany (BELLA study), 2014-2017

**Acronym: BELLA** - **BE**fragung zum see**L**ischen Woh**L**befinden und Verh**A**lten

**Implementation:** University Medical Center Hamburg-Eppendorf

**Aim:** Providing reliable information on the mental health and health-related quality of life of children, adolescents and young adults in Germany and the possibility for trends and longitudinal analyses.

**Survey design**: Combined online cross-sectional and cohort study

**BELLA cross-sectional study**

**Population:** Children and adolescents with permanent residence in Germany

**Sampling:** BELLA study participants were randomly selected from the cross-sectional sample of KiGGS Wave 2 (registry office sample). An invitation to participate in the BELLA study required prior participation in KiGGS Wave 2.

**Age range:** 7-17 years

**Sample size:** Approximately 1,400 participants

**BELLA cohort study**

**Sampling:** Renewed invitation of all participants in the BELLA baseline study (2003-2006) and BELLA Wave 3 (2009-2012) willing to take part again

**Age range:** 10-29 years

**Sample size:** Approximately 2,100 participants from the baseline survey and Wave 3

**Survey period:** November 2014-October 2017

More information in German is available at www.bella-study.org

**Figure 1:**
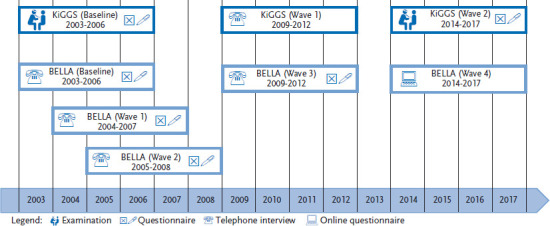
BELLA study data collection Own figure

KiGGS and BELLA baseline data were collected between 2003 and 2006, followed by two subsequent BELLA study waves (Wave 1: 2004-2007, Wave 2: 2005-2008). Two further BELLA Waves took place in parallel with KiGGS Wave 1 and KiGGS Wave 2 (BELLA Wave 3: 2009-2012, BELLA Wave 4: 2014-2017) (Figure 1). BELLA Wave 4 for the first time applied a dynamic measurement instrument (computer adaptive test, CAT) to monitor health-related quality of life among children and adolescents (Kids-CAT) [9]. Therefore, the BELLA study was able to provide up-to-date reference data for a general population sample to standardise the Kids-CAT.

## 2. Methodology

### 2.1 Study design and sampling

The BELLA Wave 4 comprises a representative sub-sample of the KiGGS study population sample. It includes roughly 3,500 children and adolescents aged 7 to 17 years, their parents as well as young adults aged 18 to 29 years. The BELLA Wave 4 cross-sectional sample is a subsample of the KiGGS Wave 2 cross-sectional sample and includes children and adolescents aged 7 to 17 years. The target population and sampling method are described in detail in the article New data for action. Data collection for KiGGS Wave 2 has been completed in this issue of the Journal of Health Monitoring. Children and adolescents were randomly drawn from the gross sample and assigned to the BELLA study during sampling for KiGGS Wave 2. An invitation to participate in BELLA Wave 4 required prior participation in KiGGS Wave 2.

The longitudinal sample of the BELLA study includes all respondents of the BELLA baseline study (2003-2006), as well as all BELLA Wave 3 (2009-2012) respondents who had in parallel participated in KiGGS Wave 1. Whether respondents had participated in BELLA Waves 1 and 2 was irrelevant.

For 7-to 10-year-olds, BELLA Wave 4 surveyed the parental assessment of the children’s mental health and health-related quality of life. For children aged 11 to 13 years, it surveyed both parental assessments and the children’s self-assessment, while adolescents aged 14 years and older were surveyed exclusively by self-assessment.

The Federal Commissioner for Data Protection has been informed and approved the study. The survey staff is bound by the provisions of the German Data Protection Act and subject to strict confidentiality. Survey data are treated with absolute confidentiality and pseudonymised prior to being saved and analysed. The survey received a positive vote from the Ethics Committee of Hamburg’s Chamber of Psychotherapists on 24 September 2014. All BELLA study respondents and/or their parents are informed about the means taken to protect their data, and provide their informed consent. Participation in the study is voluntary and respondents can cancel their participation at any time without having to give reasons.

The scientific evaluation of BELLA data relies on statistical tools to analyse and visualize frequencies and correlations for larger groups of respondents only (no analyses of individual cases). In no case is data used commercially or made available to health insurance funds and insurances.

### 2.2 Assessment methods and testing instruments

For the first time, the forth Wave of the BELLA study was conducted as a purely online-based survey. Respondents were able to fill out the questionnaire using devices such as smartphones, PCs or laptops. After consenting to being contacted by members of the BELLA team, respondents received a letter containing the consent forms and data privacy statements. Children and adolescents aged under 18 years required a written consent by at least one legal guardian. Additionally, adolescents aged 14 years and older signed their own consent form. Respondents aged 18 years and older as well as parents were able to provide their consent either in writing or electronically. Children and parents received their login details for the online questionnaire separately. All respondents received a unique user name and password to access the BELLA study at www.ichbingefragt.de. The questionnaire for young children required approximately 5 to 10 minutes to fill out; the slightly longer questionnaire for adolescents and adults required 15-20 minutes. On request, respondents without internet access were provided with a paper-based questionnaire by mail.

**KiGGS Wave 2**

Second follow-up to the German Health Interview and Examination Survey for Children and Adolescents

**Data owner:** Robert Koch Institute

**Aim:** Providing reliable information on health status, health-related behaviour, living conditions, protective and risk factors, and health care among children, adolescents and young adults living in Germany, with the possibility of trend and longitudinal analyses.

**Study design**: Combined cross-sectional and cohort study conducted as an examination and interview survey

**KiGGS cross-sectional study**

**Population:** Children and adolescents with permanent residence in Germany

**Sampling:** Samples from official residency registries - randomly selected children and adolescents from the 167 cities and municipalities covered by the KiGGS baseline study

**Age range:** 0-17 years

**Sample size:** Approximately 15,000 participants

**KiGGS cohort study**

**Sampling:** Re-invitation of everyone who took part in the KiGGS baseline study (2003-2006; aged between 0 and 17 at that time) and who was willing to participate in a follow-up

**Age range:** 10-29 years

**Sample size:** Approximately 10,000 follow-up participants

**Survey period:** September 2014-August 2017

**Modules:** BELLA, EsKiMo, GerES, KiESEL, MoMo

More information is available at www.kiggs-studie.de/english

The Wave 4 is the first Wave of the BELLA study – and presumably the first ever general German population-based sample – to use a dynamic survey method (a computer adaptive test; CAT). Based on precalculated item parameters and respondents’ previous answers CATs customise the selection of follow-up items, facilitating precise data collection on specific items [10]. Compared with conventional questionnaires (static forms of data collection), CATs are characterised by greater measurement precision and a reduced number of items. The realisation of BELLA Wave 4 is based on the experiences made with previous KiGGS and BELLA study waves, cooperation with the US Patient-Reported Outcomes Measurement Information System (PROMIS project) [11], as well as self-developed CAT instruments. The following sections present the BELLA Wave 4 measurement instruments (Table 1).

#### Health-related quality of life

**Figure 2:**
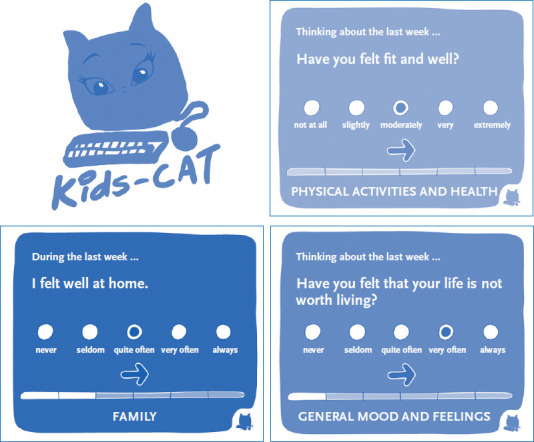
Kids-CAT in the online questionnaire (sample items) Source: [27]

The BELLA Wave 4 applied the Kids-CAT to survey child and adolescent health-related quality of life. The Kids-CAT tool, developed by the authors of this article, measures self-reported health-related quality of life based on five items banks (physical well-being, psychological well-being, parent relations, social support & peers as well as school well-being) [9] (Figure 2). For the first time, BELLA Wave 4 also integrated the proxy version (static) of Kids-CAT to survey the parents’ perspective. The KIDSCREEN-27 questionnaire [12], the SF-12 questionnaire [13] and the SF-36 questionnaire [14] were used as supplementary instruments. The KIDSCREEN is a recognised instrument to survey health-related quality of life among children and adolescents aged 8 to 18 years. The SF-36 and its short form, i.e. the SF-12, are internationally the most widely used instruments to assess the quality of life both among adolescents aged 14 years and older and among adults; it targets to provide psychometrically sound data to survey the BELLA cohort’s transition to adult age. In addition, the item banks developed by the US-based collaborative research project PROMIS [11, 23] to survey subjective well-being, family relations, physical activity, relations with peers and global health were used as validated short questionnaires. The BELLA study’s parallel use of European and American instruments allows to psychometrically test and systematically compare the applied measurement tools and constructs. In the long term, they can provide important contributions towards an international standardisation of measurement instruments to assess health-related quality of life and/or well-being.

#### Mental health problems

Child and adolescent mental health was surveyed via the Strengths and Difficulties Questionnaire (SDQ) [16, 17] used in KiGGS. For respondents aged 18 years and older, the BELLA Wave 4 used the Composite International Diagnostic-Screener (CID-S) [19], an established adult mental health screening instrument, as well as SCL-S-9 [20], a shortened form of Derogatis’ (1977) SCL-90-R symptoms check list. SCL-S-9 comprises 9 items and is used to survey mental health problems of adults. All applied psychometric measurements are established and validated mental health instruments.

To survey symptoms of depression in children and adolescents, the Center for Epidemiological Studies Depression Scale, Child (CES-DC) [25] was used. The PROMIS initiative has fostered the development of pediatric item banks for depression. These were translated into German for the BELLA study, and the shortened forms were used across all age groups. In the long term, projects focused on developing an age-independent CAT to assess symptoms of depression will be able to build on BELLA study data. A corresponding CAT for adults (D-CAT) is already available [26].

#### Mental health care use

The items used to assess mental health care were developed based on the validated tools already available. They were adapted to the specific needs of this research project. Besides surveying the psychiatric/socio-psychiatric/psycho-therapeutic, psychological or socio-pedagogic care that respondents had used, and how happy they had been with the treatment received, the tool also assessed possible treatment needs as well as barriers that prevented people from accessing treatment.

## 3. Discussion and outlook

The BELLA study supplements the KiGGS survey on mental health and health-related quality of life among children, adolescents and young adults in Germany. The BELLA study design allows data to be collected that can be used both to define the prevalence of mental disorders, analyse the corresponding developments over time and identify the determinants (risk and protective factors) linked to these developments, and to systematically assess the use of health care services during childhood and adolescence [28-31].

The BELLA study also analyses health inequalities, i.e. the differences in mental health and health-related quality of life among children and adolescents depending on their social background. The analysis of health inequalities bears a great potential to develop approaches for prevention and intervention at the level of those immediately affected (family and social environment), but also at the level of society in general (health and education system).

BELLA Wave 4 is conducted 11 years after the baseline study and therefore provides data on respondents during their passage from childhood through adolescence to young adulthood. The discussion of such transitions (for example transitions between educational institutions) and the related changes in the way people assess their health-related quality of life and mental health provide important developmental and psychological insights into childhood, adolescence and young adulthood.

In terms of methodology, the BELLA study offers an innovative and forward-thinking approach to measuring mental health and health-related quality of life across all ages. Dynamic measurement instruments such as CATs can be used in population-based cohort studies as well as in clinical practice.

As regards actual fieldwork, the online survey has proven to be highly effective both in terms of time and staff. Respondents could fill out the BELLA questionnaire on a device of their choice, ‘around the clock’, and without having to make an appointment. Respondents had easy access to the questionnaire via automatically generated user IDs, and the approach complies with data protection provisions. Data collection is set to end in autumn 2017, so initial results can be expected for the spring of 2018.

Parties interested in using BELLA study data for academic research are welcome to write to the study directors providing an outline of the planned project.

## Key statements

- The BELLA study is the KiGGS supplementary mental health module.
- The fourth wave (BELLA Wave 4) provides data for longitudinal analyses of health-related quality of life and mental health problems.
- BELLA Wave 4 relies on dynamic measurement tools to produce user-friendly and precise data on the mental health of children, adolescents and adults.
- BELLA Wave 4 data was collected online-only for the first time.

## Figures and Tables

**Table 1:** BELLA Wave 4 measurement instruments Own table